# Characterization of Reduced and Surface-Modified Graphene Oxide in Poly(Ethylene-*co*-Butyl Acrylate) Composites for Electrical Applications

**DOI:** 10.3390/polym11040740

**Published:** 2019-04-24

**Authors:** Carmen Cobo Sánchez, Martin Wåhlander, Mattias Karlsson, Diana C. Marin Quintero, Henrik Hillborg, Eva Malmström, Fritjof Nilsson

**Affiliations:** 1 KTH Royal Institute of Technology, School of Engineering Sciences in Chemistry, Biotechnology and Health, Department of Fibre and Polymer Technology, SE–100 44 Stockholm, Sweden; carmencs@kth.se (C.C.S.); wahla@kth.se (M.W.); mekarls@kth.se (M.K.); dcmq@kth.se (D.C.M.Q.); mavem@kth.se (E.M.); 2 ABB Power Technology, SE–721 78 Västerås, Sweden; henrik.hillborg@se.abb.com

**Keywords:** field grading nanocomposites, non-linear resistivity, reduced graphene oxide (rGO), HVDC, SI-ATRP surface modification

## Abstract

Promising electrical field grading materials (FGMs) for high-voltage direct-current (HVDC) applications have been designed by dispersing reduced graphene oxide (rGO) grafted with relatively short chains of poly (*n*-butyl methacrylate) (PBMA) in a poly(ethylene-*co*-butyl acrylate) (EBA) matrix. All rGO-PBMA composites with a filler fraction above 3 vol.% exhibited a distinct non-linear resistivity with increasing electric field; and it was confirmed that the resistivity could be tailored by changing the PBMA graft length or the rGO filler fraction. A combined image analysis- and Monte-Carlo simulation strategy revealed that the addition of PBMA grafts improved the enthalpic solubility of rGO in EBA; resulting in improved particle dispersion and more controlled flake-to-flake distances. The addition of rGO and rGO-PBMAs increased the modulus of the materials up to 200% and the strain did not vary significantly as compared to that of the reference matrix for the rGO-PBMA-2 vol.% composites; indicating that the interphase between the rGO and EBA was subsequently improved. The new composites have comparable electrical properties as today’s commercial FGMs; but are lighter and less brittle due to a lower filler fraction of semi-conductive particles (3 vol.% instead of 30–40 vol.%).

## 1. Introduction

Polymeric nanocomposites, i.e., polymers with nanofillers incorporated into the matrix, have been intensively studied during the last decades. The extremely large specific surface area of all nanofillers can be exploited to improve many material properties. Traditional composites are typically designed for improving the mechanical properties of the material, but nanocomposites are more versatile; for instance, the thermal and electrical properties are also influenced by the addition of nanofillers such as graphene, ZnO, or Al_2_O_3_ [1,2]. 

A good dispersion of the nanoparticles is generally required to maximize the effective particle surface area and to achieve a homogenous material with few detrimental material flaws. Ultra-sonication of the nanoparticles can be performed prior to mixing to break-up particle agglomerates and facilitate good particle dispersion. Efficient mechanical mixing during the nanocomposite manufacturing is also desired. The dispersion is further influenced by the volume filler fraction of particles and by the predisposition of the particles to self-assemble and form agglomerates within the matrix; nanoparticles are typically prone to aggregate due to their large surface area. Moreover, if hydrophilic inorganic nanoparticles, such as SiO_2_, Al_2_O_3_, or ZnO, are introduced directly into a hydrophobic polymer matrix, the particle miscibility and the adhesion between the particles and the matrix can easily become unsatisfying. Even though a proper surface modification of the nanofillers can significantly improve the dispersion [2,3], it is still a major challenge for scientists and material producers to obtain completely controlled dispersions.

Field grading materials (FGMs) are defined as materials with a resistivity depending on the applied electrical field (*E*-field). In particular, non-linear resistive FGMs have an electrical response that rapidly switches from insulating to conducting when the *E*-field is increased above a threshold level. Such materials can be used to minimize the risk of electrical breakdown due to local *E*-field enhancements in crucial electrical accessories such as high-voltage direct-current (HVDC) cable joints and cable terminations. Currently available nonlinear FGMs are typically rubber-based, percolated composites filled with approximately 30–40 vol.% particles [4,5,6]. The high particle filler fractions may result in unnecessarily heavy and brittle composites. 

Graphene, graphene oxide (GO), and (partially) reduced graphene oxide (rGO) have interesting electrical properties [2,7,8,9,10,11] and have therefore attracted attention as promising fillers for the next generation of non-linear resistive FGMs [2,7,8,9,10,11]. For instance, the addition of 2.5 vol.% partially reduced GO into a silicone rubber matrix and resulted in a material with non-linear resistivity [12]. In this study, the abbreviation rGO is used, for simplicity, both for partially and completely reduced GO. The reduction of GO, either by thermal treatment [13] or chemical procedures [14], reduces the oxygen fraction of the nano-flakes. A higher reduction degree of the rGO therefore corresponds to a higher conductivity of the rGO flakes. The electrical properties of a completely reduced rGO are thus close to the properties of pure graphene. When GO is thermally reduced in air at 120 °C, as in this study, the removed oxygen atoms mainly come from C–O and C–OH bonds, while the oxygen from C=O and O=C-OH groups remain nearly unaffected [13].

In a recent study [2], the addition of 2 vol.% poly(*n*-butyl methacrylate) surface-modified rGO (rGO-PBMA) in a poly(ethylene-*co*-butyl acrylate) (EBA) matrix enabled a pronounced non-linear resistivity. Three different polymer graft lengths (10, 50, and 70 kg mol^−1^) were used to improve the compatibility between the rGO and the EBA matrix and to avoid aggregation of the filler. The rGO-PBMA with the shortest polymer grafts showed a rapid and sharp non-linear resistivity above the *E*-field threshold. The resistivity results were explained by three observations: (1) since the composites were isotropic with filler fractions locally higher than the predicted electrical percolation threshold, longer graft lengths resulted in longer interparticle distances, (2) the average interparticle distance was close to the maximum charge-carrier hopping distance, and (3) an increased *E*-field increased the hopping distance. Therefore, a field-grading non-linear resistivity was expected with increasing electrical field, which was also observed. 

This work is an extension of the previous rGO-PBMA/EBA study [2]. A slightly updated nanocomposite preparation technique is here utilized, enabling a higher degree of control and predictability of the composite resistivity. EBA-based nanocomposites with 2, 3, and 4 vol.% of rGO and rGO-PBMAs, respectively, were prepared by solvent casting in *p*-xylene. Three comparatively short graft-lengths of 3.0, 4.5, and 9.2 kg/mol were chosen to further decrease the inter-flake distance compared to our previous study. The morphology and resistivity of the composites were analyzed both with SEM, resistivity measurements, and Monte-Carlo computer simulations in order to reveal how the non-linear composite resistivity is influenced by the dispersion and orientation of the rGO flakes. The aim of this work was to develop light and stable FGMs, with tuneable electrical nonlinearities and improved mechanical properties, for HVDC applications. 

## 2. Experimental Section

### 2.1. Materials

Poly(ethylene-*co*-butyl acrylate) pellets (EBA, Mw = 205 kDa, 27% BA) were supplied by Borealis AB (Stenungssund, Sweden). Freeze-dried GO was purchased from Abalonyx (Oslo, Norway) and homogenized in ethanol:water mixtures (1:1 *v*/*v*), purified and partially reduced by thermal reduction in air at 120 °C for 20 h prior to use or surface modification. Chemicals were used as received from Sigma-Aldrich unless stated otherwise; (3-aminopropyl)triethoxysilane (APTES, 98%), n-butyl methacrylate (BMA, 97%), 2-bromoisobutyryl bromide (α-BiB, 98%), ethyl α-bromoisobutyrate (EBiB) (98%), copper(I) bromide (Cu(I)Br, 98%), copper(II) bromide (Cu(II)Br2, 99%), 4-(dimethylamino) pyridine (DMAP, 99%), triethylamine (TEA, 99%), and 1,1,4,7,10,10-hexamethyltriethylenetetramine (HMTETA, 97%). Butyl methacrylate (BMA, 96%) was destabilized prior to use by passing it through a column of Al_2_O_3_ (neutral). Deionized water, ethanol (EtOH, 96%), tetrahydrofuran (THF, analytical grade), dichloromethane (DCM, analytical grade), methanol (HPLC-grade), diethyl ether (HPLC-grade), *p*-xylene, and toluene (HPLC-grade) were used without further purification. 

### 2.2. Procedures to Modify GO: Thermal Reduction, Silanization, Attachment of Initiator, and Surface-Initiated Atom-Transfer Radical Polymerization (SI-ATRP) of PBMA (rGO, rGO-Silanized, rGO-Initiated and rGO-PBMAs)

The procedures used to obtain rGO-Silanized, rGO-Initiated, and rGO-PBMAs are published elsewhere [2] and only summarized below.

GO (500 mg) was added to a round bottom flask, with EtOH (150 mL), equipped with a magnet stir bar. Deionized water (150 mL) was added and the mixture was ultrasonicated for 5 min and thereafter stirred overnight. The suspension was purified with EtOH:water (1:1 *v*/*v*) twice, by centrifugation (20 min at 21,000 g) and ultrasonication (5 min) cycles. Finally, the GO was freeze dried and thermally reduced in an oven at 120 °C for 20 h, obtaining partially reduced graphene oxide (rGO). This partial reduction process decreases the oxygen content from approximately 31.5% (GO) to 19.7% (rGO) [13]. 

In order to obtain rGO-Silanized, EtOH (225 mL) was added to rGO (500 mg). Deionized water (100 mL) was added to the suspension and the mixture was ultrasonicated (5 min in bath and 30 s, 30% amplitude with 8/2 sec on/off pulses). Then, (3-Aminopropyl)triethoxysilane (APTES, 15 mL, 200 mmol) was added dropwise to the suspension at room temperature (RT), and subsequently the reaction continued under reflux at 80 °C overnight. The final rGO-Silanized suspension was purified by three centrifugation and ultrasonication cycles in EtOH:water (2:1) and finally twice in THF.

To create rGO-Initiated (~500 g) suspended in THF (60 mL), α-bromoisobutyryl bromide (0.97 g, 4.2 mmol α-BiB), trimethylamine (TEA, 0.60 g, 4.9 mmol), and 4-(dimethylamino)pyridine (DMAP, 5 grains) were added and left on a shaking table overnight. A small amount of EtOH was added to quench the reaction before purification of rGO-Initiated. The final material was purified by successive centrifugation–homogenization (ultrasonication) cycles in THF:EtOH, THF, DCM, and finally twice in toluene. The final rGO-Initiated was suspended in toluene (20 mL).

Surface-initiated atom-transfer radical polymerization (SI-ATRP) was used to graft PBMA polymer chains from the rGO-Initiated surfaces. rGO-Initiated (70 mg), toluene (15 mL), destabilized n-butyl methacrylate (BMA, 62.8 mmol, 8.93 g), 1,1,4,7,10,10-hexamethyltriethylenetetramine (HMTETA, 24.5 µL, 90 µmol), and ethyl α-bromoisobutyrate (EBiB, 11.8 µL, 81 µmol) were added to a round bottom flask equipped with a stirrer and degassed by 2 vacuum/argon cycles (5 + 5 min). Subsequently, copper(I) bromide (CuBr, 10 mg, 72 µmol) and copper(II) bromide (CuBr_2_, 4.0 mg, 18 µmol) were added and two extra degassing cycles performed. The reaction was placed in an oil bath preheated to 60 °C and left to react for 2, 4, or 6 h, to yield rGO-PBMA-3K, rGO-PBMA-4.5K and rGO-PBMA-9K. The rGO-PBMAs were purified by successive centrifugation and ultra-sonication cycles in THF (three times), using the first supernatant to recover the free PBMA formed from the sacrificial initiator during the polymerization and characterized by DMF-SEC.

FT-IR and TGA were used to characterize the rGO, rGO, rGO-Silanized, rGO-Initiated, and rGO-PBMAs.

### 2.3. Nanocomposite Formation from rGO-PBMAs and the EBA Matrix

The rGO and rGO-PBMAs were used in 2, 3, and 4 vol.% (4, 6, and 8 wt %) in an EBA matrix. First, the required amounts of filler were weighed in vials and dispersed in *p*-xylene (5 mL) using the ultrasonication bath (10 min). EBA (200 mg) was weighed in a vial equipped with a magnetic stirrer, and subsequently added to the rGO dispersion. The vial was then placed in an oil bath preheated to 115 °C, and the reaction mixture was stirred for 1 h. The mixture was poured in a flask and left to dry at 90 °C overnight in an oven, followed by another two days drying at 60 °C. The resulting nanocomposites were hot-pressed into discs (3.4 cm diameter, 148 ± 7 µm thick, 150 °C, 145 kN, 20 min). Due to the highly polished surfaces of the press plates, the largest observed thickness variation within an individual disc was only 2.9%. The samples were finally characterized with SEM and resistivity measurements.

### 2.4. Instrumentation and Characterization Methods

Size exclusion chromatography (SEC) was performed using dimethylformamide (DMF) (0.2 mL·min^−1^) as the mobile phase at 50 °C, using a TOSOH EcoSEC HLC-8320GPC system equipped with an EcoSEC RI detector and three columns (PSS PFG 5 μm; Microguard, 100 Å, and 300 Å) (*M*_W_ resolving range: 300–100,000 Da) from PSS GmbH. A conventional calibration method was created using narrow linear poly(methyl methacrylate) (PMMA) standards. Corrections for flow rate fluctuations were made using toluene as an internal standard. PSS WinGPC Unity software version 7.2 was used to process data.

Fourier transformation infrared spectroscopy, FT-IR, was performed with a Perkin-Elmer Spectrum 2000 FT-IR equipped with a MKII Golden Gate, single reflection ATR System (from Specac Ltd., London, U.K.). The ATR-crystal was a MKII heated Diamond 45° ATR Top Plate. 

Thermogravimetric analysis (TGA) was performed with a TA Instruments Hi-Res TGA 2950 analyzer (TA Instruments, Newcastle, Ireland) under nitrogen flow. The heating rate was 10 °C/min and the experiments were performed from 40 to 700 °C. Two measurements were done for each material composition and the average was used in the plots. 

Differential scanning calorimetry (DSC) was performed with a DSC 1 from Mettler-Toledo. Samples of 5–10 mg were measured through a cycle of heating–cooling–heating, with a starting temperature of −30 °C and up to 160 °C at a heating/cooling rate of 10 °C/min. 

A Hitachi S-4800 field emission scanning electron microscope was used to characterize the fractured film surfaces. 

Tensile tests of the composites were performed using a universal materials testing machine Instron 5944 (Instron., Norwood, MA, USA) equipped with a 500 N load cell. The samples were conditioned at 23 °C under controlled relative humidity of 50 RH% for at least one week prior to the tensile test. The samples were cut in strips (25 × 5 mm) and tested at a 50%/min strain rate. All the mechanical data reported were obtained from at least three specimens for each sample and the means and standard deviations were reported.

Resistivity measurements perpendicular to the film direction of the approximately 150 µm thick pressed plates were conducted in an oven (Binder FED 115) for maintaining a controlled temperature of 25 °C and additional shielding. A high voltage electrode of stainless steel and a guarded measurement electrode system of brass with a 20 mm diameter of the inner electrode were used. The electric field was increased with polarizing/depolarizing cycles of 12 min from 0.2 to 8 kV mm^−1^. The high voltage supply was a HCP-35-12500, FuG Elektronik (Schechen, Germany) and a 6517B electrometer, Keithley (Cleveland, Ohio, USA) was used to measure the current through the sample. The thickness of the individual specimen was used when converting the measured current to resistivity. Only one measurement was reported for each material composition, but additional replicate measurements were also performed, indicating good reproducibility.

## 3. Results and Discussion

### 3.1. Characterization of the rGO Surface Modifications Using TGA, FT-IR, and SEC

**Figure 1:**
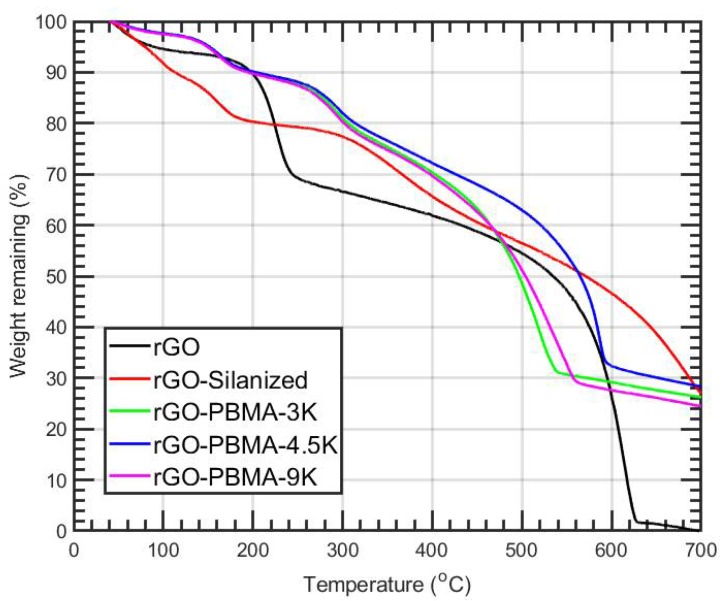
TGA thermograms of reduced graphene oxide (rGO), after silanization (rGO-Silanized), and after subsequent polymer grafting (rGO-PBMA). PBMA = poly(*n*-butyl methacrylate).

The TGA thermograms for all the rGO modifications are shown in Figure 1. In agreement with the previous results [2], thermal stabilization and higher degradation temperatures (from 160 to 250–300 °C) were observed for the rGO-PBMAs upon silanization and addition of PBMA, indicating that grafting had occurred. Pure PBMA became completely decomposed around 400 °C [11].

A reduction of carbonyl and hydroxyl groups after the rGO silanization can be observed by FT-IR (Figure S1, Supporting Information). The peaks at ca. 3500 cm^−1^ (hydroxyl groups and moisture) and 1750 cm^−1^ (carbonyl groups) decreases, while an additional peak appears around 1550 cm^−1^, corresponding to the N–H bond from the APTES. The subsequent addition of PBMA was, however, difficult to capture with FT-IR (Figure S2, Supporting Information). No distinct increase in the carbonyl peak intensity (at 1750 cm^−1^) was observed, indicating a relatively low quantity of PBMA present in the rGO-PBMA samples. 

SEC was used to assess the molar mass and molar mass distribution of the PBMA formed from the sacrificial initiator as a function of reaction time. It is assumed that the grafted polymer has similar characteristics as the unbound polymer [15]. The SEC traces are shown in Figure S3 (Supporting Information), resulting in 3.0, 4.5, and 9.2 kg/mol for reaction times at 2, 4, and 6 h. The PDI values varied between 1.1 and 1.2. The combination of fairly low PDI values and a distinct PBMA chain length increase with reaction time indicates that the polymerizations were successfully controlled.

### 3.2. Characterization of the Nanocomposites Using TGA, DSC, SEM, and Tensile Tests

Nanocomposites containing 2, 3, and 4 vol.% of rGO and rGO-PBMAs were blended with EBA and denoted rGO-*Y* or rGO-PBMA-*X*-*Y*, respectively, where *X* is the PBMA length and *Y* is the vol.% of nanofiller present in the system. 

**Figure 2:**
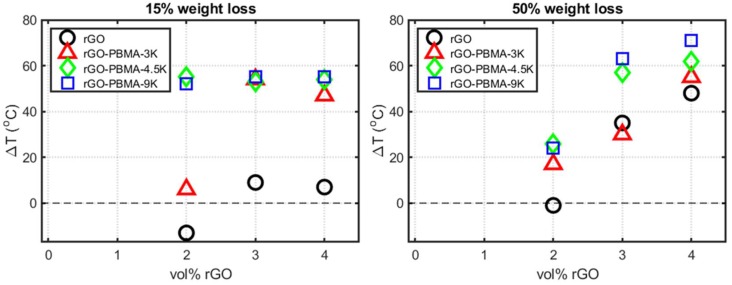
Thermal stability increase versus nanofiller fraction at (**left**) 15% weight loss and (**right**) 50% weight loss. The data are extracted from TGA thermograms.

TGA thermograms for all the nanocomposites are shown in Figure S4 (Supporting Information). For weight losses of 15% and 50%, the nanocomposite and EBA temperatures *T* and *T*_0_ were extracted from the TGA thermograms. The resulting temperature differences, Δ*T* = *T* − *T*_0_, were subsequently plotted as a function of filler fraction in Figure 2 such that positive Δ*T* values correspond to increased thermal stability. As observed in Figure 2a, the temperatures for 15% weight loss were approximately 50 °C higher for the rGO-PBMA-based nanocomposites than for the EBA reference and the nanocomposites with unmodified rGO. We suggest that the inherent thermal stability of rGO increases upon grafting with PBMA. At 50% weight loss, a nearly linear increase in thermal stability with increasing rGO content was observed for all nanocomposites (Figure 2b). The thermal stability increase was in the range 0–30 °C at 2 vol.% and 45–75 °C at 4 vol.% rGO. The remarkable improvements in thermal stability with PBMA grafting and with increasing rGO fractions can potentially be explained by the inherent ability of rGO to impede penetrant gas diffusion of vaporized molecules in the composite [2]. An increased rGO filler fraction would thus result in decreased penetrant gas diffusivities and thus in an increase in thermal stability. Also, the presence of PBMA grafts reduces the diffusivity in EBA nanocomposites, thus incrementing the effect [2].

DSC data for all the nanocomposite films are presented in Table S1 (Supporting Information) and some examples of the first heating thermograms are plotted in Figure S5 (Supporting Information). The peak (around 42.5 °C) observed during the first heating is probably an effect of the compression molding. A certain level of entropy relaxation may arise because of the space constraints during hot-pressing. Since both the peak position and the integral values are approximately equal for all the samples, it was concluded that the crystallinities were relatively insensitive to the addition of rGO or rGO-PBMA.

**Figure 3:**
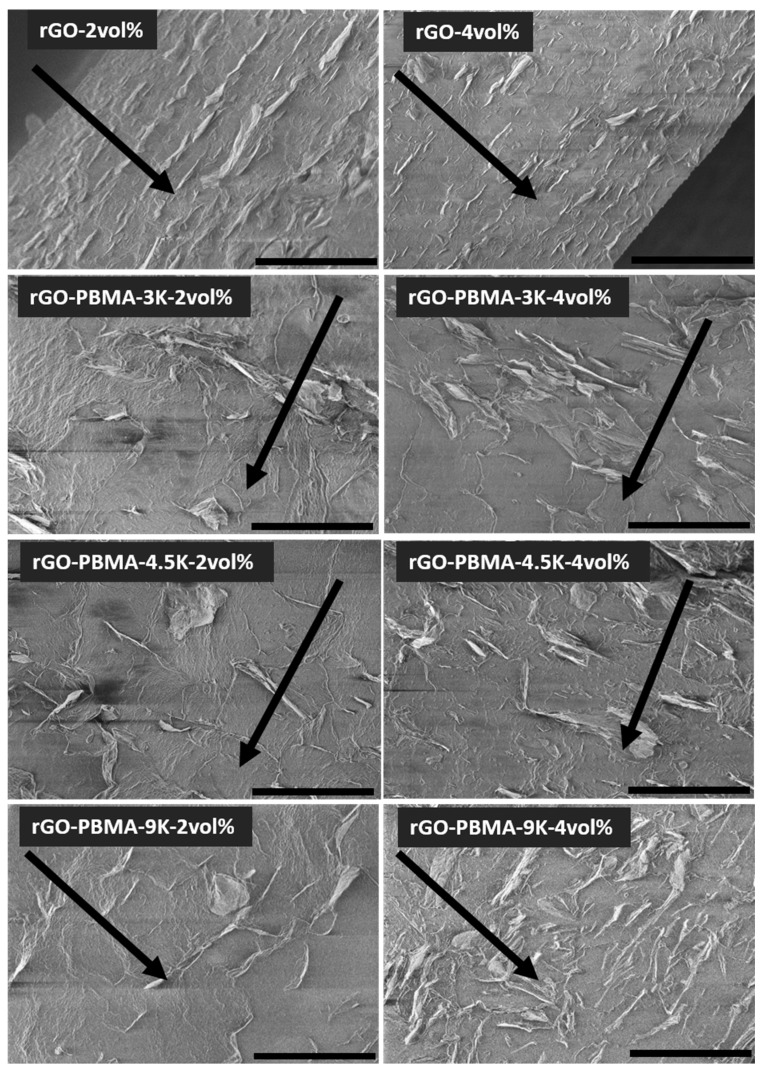
SEM micrographs of cross-sections of the nanocomposites, the arrows indicate the film direction (i.e., perpendicular to the pressing direction). The unmodified rGO sheets are clearly aligned in the film direction (top row). The polymer-grafted rGO gradually develop a more random direction with increasing length of the polymer brushes. All scale bars are 20 µm.

SEM was used to study the morphology of nanocomposite cross-sections containing 2 and 4 vol.% fillers (Figure 3). The rGO and rGO-PBMA flakes showed slight agglomeration in all samples, but more importantly, they showed directionality along the film direction (parallel to the pressing direction). This directionality starts to disappear in the rGO-PBMA-9K-4 vol.% composite, where a more random and connected structure was observed. The nearly isotropic morphology indicates that the flakes and clusters are entropically mixed within the matrix, and thus, unaffected by the applied pressing force and temperature. 

**Figure 4:**
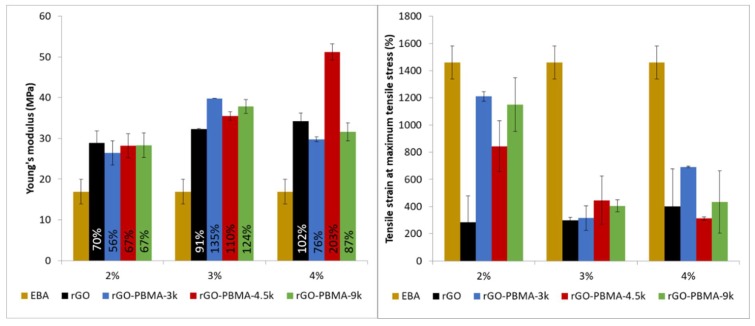
Young’s Modulus (left) and tensile strain at maximum tensile stress (right) of the rGO-nanocomposites.

The mechanical properties of the rGO-based nanocomposites were assessed by tensile testing and compared to the neat EBA matrix (Figure 4). Young’s modulus was found to increase by 70%, 91%, and 102% for the 2%, 3%, and 4% nanocomposites by the mere addition of neat rGO to the matrix [16,17,18,19,20,21]. At the same time, the nanocomposites showed a significant decrease in tensile strain, from approximately 1400% to 400%, as a consequence of failure points related to the addition of 3-4% nanofiller and the appearance of nanofiller-clusters or even percolated networks. Interestingly, the rGO-PBMAs-2% nanocomposites, with similar Young’s modulus values as their rGO counterpart, showed a lower decrease in tensile strain, between 1200% and 900%, which is similar to the neat EBA. This is in agreement with previous observations reported in the literature and attributed to interactions between the polymer chains grafted from the filler and the matrix, resulting in improved adhesion between the components and a reduced and/or delayed failure by breakage of the nanocomposites [19,22]. This enhanced compatibility between the matrix and the grafted nanofiller leads to a slight increase in Young’s modulus, as also has been reported in the literature [20,21,23]. In our study, the rGO-PBMA-4.5k-4% nanocomposite shows an astonishing increase in Young’s modulus of 200%, while it decreases for rGO-PBMA-3k-4% and rGO-PBMA-9k-4%. It can be argued that the intermediately sized PBMA-chain might be long enough to interact with the matrix polymer (EBA) but not with chains attached to the same nanofiller, while the longer PBMA chains might be more prone to interact with neighboring grafts, being detrimental for the adhesion between the two components [24]. This has been observed in epoxy-based nanocomposites with polymer-grafted GO carrying two different polymer lengths [25]. Interestingly, the rGO-PBMA-3k and rGO-PBMA-9k have similar Young’s modulus vaules. This can be attributed to a higher number of nanofillers in the rGO-PBMA-3k compared to the rGO-PBMA-9k with the same wt % fraction. Even though the quantities of polymer grafted from rGO are very small, they still affect the overall performance and should therefore be taken into account in order to understand the obtained results. Thus, even if PBMA-3k is long enough to interact with the matrix and improve the mechanical properties, an increasing number of nanofillers would lead to an increase of breakage points and agglomeration, with a subsequent decrease in Young’s modulus upon addition of nanofiller. 

### 3.3. DC Resistivity Measurements of the Nanocomposites

**Figure 5:**
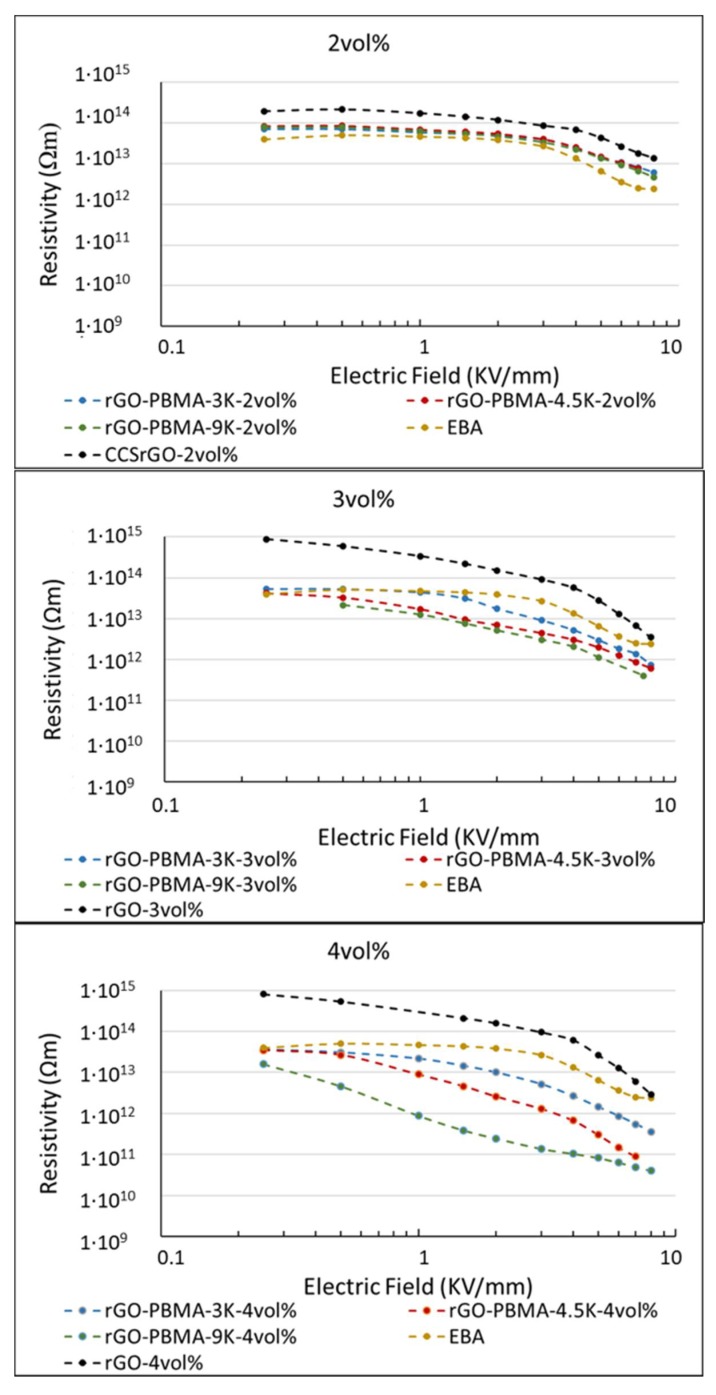
Resistivity measurements for the 2, 3, and 4 vol.% nanocomposites.

The DC resistivity at an *E*-field between 0.2 and 8 kV mm^−1^ of neat EBA and EBA-nanocomposites containing rGO and PBMA-rGO was characterized at room temperature (Figure 5). The apparent non-linear slope in resistivity at higher *E*-fields (>3 kV mm^−1^) of neat EBA was an effect of the current having not fully reached the steady-state value, resulting in an apparent decrease in resistivity. It is interesting to note that the EBA composites filled with 2 vol.% rGO exhibited a slightly higher resistivity (2 × 10^14^ Ωm) than the neat EBA (1 × 10^14^ Ωm). This effect, which has been exploited in the development of insulating composites [2,12,26,27], has been attributed to the trapping of charge carriers (i.e., electrons, holes, ions, and polar molecules such as water) on the rGO interfaces [2,12,26,27].

In contrast to the EBA nanocomposites with 2 vol.% unmodified rGO, the corresponding nanocomposites with 2 vol.% PBMA-rGO all exhibited a similar resistivity as the neat EBA, which may be explained by the reduced amount of interfacial polar groups, and hence the more hydrophobic character of the PBMA grafted rGO [2]. Since all 2 vol.% composites also exhibited similar behavior as net EBA, although at different resistivities, one may argue that these composites also exhibit a linear, rather than a non-linear, resistivity.

The EBA filled with 3 vol.% polymer-grafted rGO exhibited a lower resistivity compared to that of neat EBA at *E* ≥ 0.5 kV mm^−1^. The difference in resistivity was further enhanced at a concentration of 4 vol.% of polymer-grafted rGO. The slope of the curves above this onset voltage corresponds to the non-linearity coefficient (α) [2,12,26,27]:

$$\alpha =1+\frac{dln\left(\sigma \right)}{dln\left(E\right)}$$

where *σ* is the conductivity of the material (i.e., the inversed resistivity) and *E* is the field strength. Insulating materials’ resistivity is independent on the applied voltage level and exhibit α = 1. At 4 vol.% concentration, the nanocomposite with the shortest polymer brush, rGO-PBMA-3k, exhibited a weak non-linear resistivity (α = 1.5) finally reaching a resistivity of 4 × 10^11^ Ω m. The material containing the intermediate length of the polymer graft, rGO-PBMA-4.5k, exhibited a distinct non-linear resistivity (α = 2.5) and a final resistivity of 1 × 10^11^ Ω m. Finally, the material containing the longest length of the polymer grafts, rGO-PBMA-9k, exhibited the highest non-linear resistivity (α = 3.2), and a final resistivity of 4 × 10^10^ Ohm m. Thus, the non-linear coefficient increased with increasing chain length of the polymer grafts. In addition, the resistivity at the higher electric fields decreased with increasing polymer grafts. Overall, the changes in resistivity were lower than expected, with only a three orders of magnitude change versus the six orders obtained in the previous study [2], using nearly equal graft lengths. The differences could potentially be explained by the differences in nanocomposite preparation, which can change the rGO dispersion within the EBA matrix. In this study, the rGO and rGO-PBMAs were dispersed in *p*-xylene at room temperature, then the EBA was added and mixed at high temperature, while in the previous study, the rGO and rGO-PBMAs were dispersed in *p*-xylene at room temperature and subsequently added to the hot EBA solution in *p*-xylene, followed by ultrasonication at room temperature. 

The nanocomposites, which were previously prepared, displayed amorphous poly(butyl acrylate) (PBA) rich areas with high rGO-PBMAs concentrations, probably as a consequence of the EBA cooling during the mixing and ultrasonication at room temperature. In this work, the processing was conducted at the EBA softening temperature (or higher), and rGO-rich containing areas were therefore not observed in SEM (Figure 3). 

**Figure 6:**
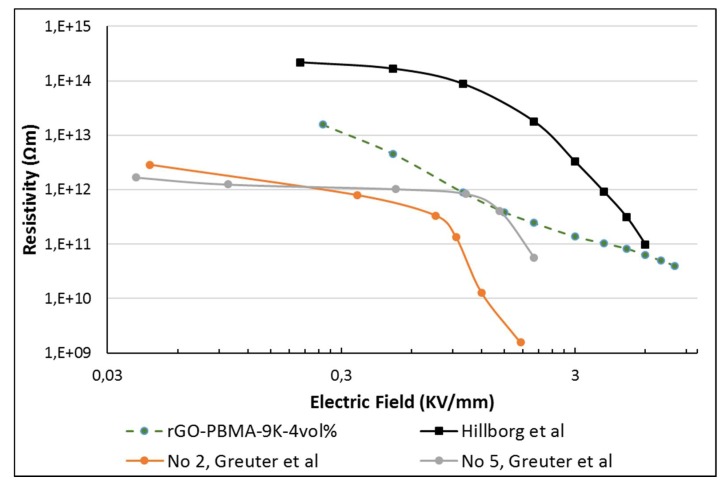
Comparison of non-linear resistivity of rGO-PBMA-9k with literature data. The commercial grade field grading materials (No. 2 and 5 in Greuter et al. [6]) exhibit a significantly lower resistivity at the lower fields, and higher non-linearity, compared to rGO-PBMA-9k. A field grading material based on 1.5 vol.% rGO in silicone rubber, reported by Hillborg et al. [24] exhibits a higher resistivity.

If the PBA-rich areas in the old study formed a continuous network, that superstructure would enable electrical percolation at a lower rGO filler fraction than otherwise needed, assuming that the rGO fillers are concentrated to the PBA network. The distances between the rGO flakes would also decrease, leading to shorter hopping distances for the charge carriers and a lower resistivity. The network hypothesis was strengthened by the observation that 2 vol.% fillers was enough to significantly alter the resistivities in the previous study, but in this study at least 3 vol.% was needed. The lower non-linear resistivities of the materials prepared in the current study (α = 1.5–3.2) are more similar to polymeric field grading materials having a homogenous distribution of semi-conductive fillers. This is shown in Figure 6, comparing rGO-PBMA-9k with literature data. In a previous study by Hillborg et al. [13], the non-linear resistivity of 1.5 vol.% rGO in silicone rubber exhibited a similar resistivity at low fields (2 × 10^14^ Ohm m), a non-linear coefficient of ~6 (at *E* > 2 kV mm^−1^), and a final resistivity of 1 × 10^11^ Ohm m. The rGO was homogenously dispersed in the amorphous PDMS matrix, achieving a percolated network at this low concentration. Two types of commercial field grading materials for HVDC cable accessory applications (No. 2 and 5 in a review by Greuter et al. [6]), based on polymers filled with a percolated network of SiC particles show a similar modest nonlinearity, α ≈ 3–6. A potential advantage of the EBA-nanocomposites containing 4 vol.% PBMA-rGO is the high resistivity at lower fields, in combination with the stable slope in non-linearity, observed at fields up to 8 kV/mm. This can be a useful property when designing resistive field grading materials, operating at higher fields, compared to the commercial grades illustrated in Greuter et al. [6]. As a complement to the network hypothesis, the effect of filler orientation was examined with Monte-Carlo simulations, using rGO aspect ratios and orientations from SEM.

### 3.4. Monte-Carlo Simulation Results for the Nanocomposite Resistivity

**Figure 7:**
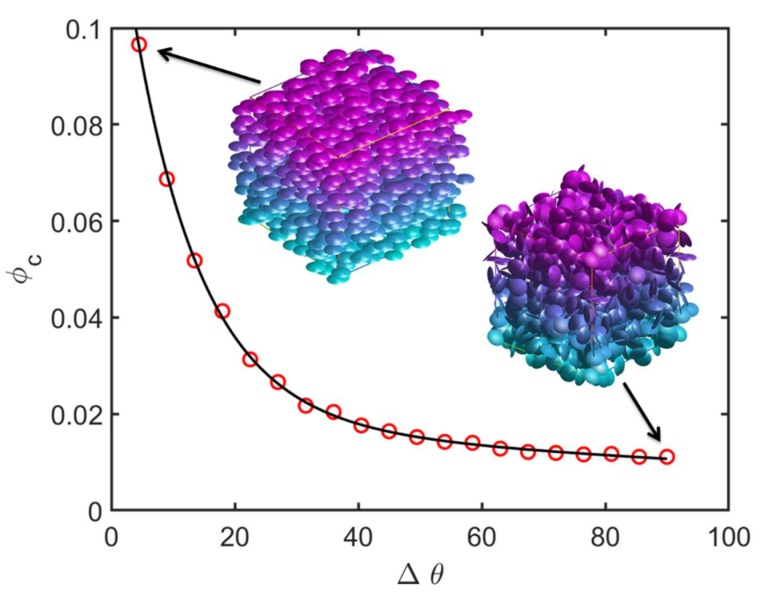
Percolation threshold φ_c_ as a function of the degree of isotropy (0 = minimum, 90 = maximum) for simulated nanocomposites with nanofillers having an aspect ratio of 100.

In order to facilitate the understanding of the experimental resistivity results, Monte-Carlo simulations were used to assess the impact of the anisotropic rGO orientations observed in the SEM micrographs for the rGO-PBMA-9K-4 vol.% sample (Figure 3). The electrical percolation threshold of different rGO composites with varied filler orientations was predicted with a percolation threshold model where the rGO flakes were modelled as soft oblate cylinders with an effective aspect ratio equal to 100, i.e., the diameter is 100 times larger than the thickness [2,28]. The aspect ratio was chosen based on measurements from SEM images [2]. In our simulations, the cylindrical fillers were added one by one into the cubical computational domain of the modelled composite until a continuous path of fillers bridged the whole domain from top to bottom. This critical filler fraction was defined as the percolation threshold (*ϕ*_c_) of the composite. Each simulation used approximately 25,000 filler particles and an average of 100 simulations was used for each reported data point. All rGO flakes were initially positioned perpendicular to the electrical field and were thereafter given a random orientation with a maximum deviation angle Δ*θ* from the initial orientation. For anisotropic composites where the flakes are nearly parallel to each other (Δ*θ* = 0°), the percolation threshold is much higher than for isotropic systems (Δ*θ* = 90°) where the particle orientation is completely random (see Figure 7). The underlying explanation we put forward is that the probability for an intersection between two flakes becomes larger when the fillers are more randomly oriented, resulting in percolated paths throughout the composite already at lower filler fractions [3,26]. Corresponding angular distributions were calculated from the SEM graphs, revealing that the experimentally measured Δ*θ* is around 10–30° for the composites with naked rGO and around 50–90° for the grafted rGOs with the longest PBMA chains. Figure 7 thus predicts that the percolation threshold of composites with naked and grafted rGO particles (with an aspect ratio of 100) should be around 3–7 vol.% and 1–2 vol.%, respectively. The experimental resistivity measurements indicate that the true percolation threshold is above 4 vol.% for composites with naked particles, and between 2–4 vol.% for those with grafted particles. The predicted percolation thresholds are thus slightly lower than those experimentally observed, but considering the uncertainty in effective rGO aspect ratios, the simulations can still explain the experimentally observed phenomena reasonably well. A slightly lower effective aspect ratio would result in a trend that is in perfect agreement with the experimental measurements. The experimentally observed decreasing resistivity with increasing graft length can most probably also be explained by the increasing degree of isotropy. 

## 4. Conclusions 

Thermally reduced graphene oxide (rGO) sheets were successfully surface modified with PBMA using SI-ATRP. PBMA chains with molecular weights 3, 4.5, and 9 kg/mol were thus grafted from silanized and initiator immobilized rGO surfaces, to obtain PBMA-grafted reduced graphene oxide (rGO-PBMA). Short polymer grafts significantly increased the thermal stability of the nano-fillers and confirmed that the modification was successful. EBA nanocomposites with rGO and rGO-PBMAs were prepared by solvent casting and subsequently hot-pressed to create films. The thermal stability of the nanocomposites increased significantly by increasing the rGO filler fraction or by adding PBMA grafts, reaching a 70 °C increase at 4 vol.% rGO-PBMA. A probable explanation is that the rGO flakes impede diffusion of volatile combustion gases from the composite and thus stabilize the material.

SEM analysis of nanocomposite cross-sections revealed nanoflake dispersions with acceptable degrees of agglomeration, although a distinct directionality perpendicular to the pressing direction was observed in all samples, except for the rGO-PBMA-9K nanocomposites. Longer graft lengths result in extended interparticle distances which, in turn, facilitate the manufacturing of composites with isotropic particle orientations where the directionality is also limited after hot-pressing. The Young’s modulus of the nanocomposites increased upon addition of rGO, being more significant for the rGO-PBMA-4.5-4 vol.%, and the strain at maximum tensile stress of the rGO-PBMAs improved significantly at low loadings, indicating that the grafting of PBMA from the rGO increases the compatibility between the filler and the EBA matrix. 

The SEM micrographs were used to measure the aspect ratios and orientations of the rGO sheets. These aspect ratios and orientations were inserted into a Monte-Carlo simulation model to predict the electrical percolation threshold as a function rGO orientation. A percolation threshold around 1–2 vol.% was predicted for nearly isotropic composites such as rGO-PBMA-9K and a corresponding threshold around 3–7 vol.% was predicted for more anisotropic composites, such as the composites with unmodified rGO. Below the threshold, the composite resistivities were expected to be similar to or higher than the EBA resistivity. Above the threshold the composite resistivities were expected to be lower and dependent on the length of the grafts separating the flakes. Field-dependent DC resistivity measurements were applied on the nanocomposites. At low filler fractions (2 wt %) the nanocomposites’ resistivities were slightly higher than that of the EBA reference, probably due to the trapping of electrons, ions, and polar molecules at the (well-separated) rGO surfaces. At higher filler fractions (3–4 vol.%), the PBMA-rGO/EBA resistivities were lower than that of the reference and decreased with increasing PBMA-graft length. Modeling suggests that an increasing PBMA length increases the interflake distances, which increases the resistivity. Simultaneously, the isotropy of the grafted rGO sheets increases, which subsequently decreases the resistivity. For sufficiently orientated rGO sheets with relatively short chains, the latter effect seems to dominate. This conclusion was supported by SEM images showing the highest isotropy in the nanocomposites with the longest PBMA-grafts.

To summarize, this study concludes that materials with *E*-field dependent resistivities can be obtained by dispersing small fractions (2–4 wt %) of PBMA-grafted rGO in EBA, which is in agreement with our previous results; the resistivity profile can be tailored towards desired applications by varying the rGO fraction and the PBMA graft length. Since these composites are lighter and have better mechanical properties than today’s field grading materials, they are promising candidates as field grading material in electrical HVDC applications. 

## Supplementary Materials

The following supporting information is available online at https://www.mdpi.com/2073-4360/11/4/740/s1, Figure S1: FT-IR spectra from the rGO and rGO-silanized samples. The peak for carbonyl bonds disappears for the rGO-silanized, appearing one more peak for the N–H bond from the APTES; Figure S2: FT-IR spectra from the rGO-Initiated and the rGO-PBMAs. Due to the low quantity of polymer in the samples, no change in the carbonyl peak could be observed; Figure S3: Overlay from the DMF-SEC samples measured from the polymer formed from the sacrificial initiator, corresponding from left to right to 2, 4 and 6 hours; Figure S4: TGA thermograms for all the nanocomposites, up to down; Figure S5: First heating thermograms for all the nanocomposites, up to dow; Table S1: DSC data for all the nanocomposites.
