# Supplementary material for: Characterization of Reduced and Surface-Modified Graphene Oxide in Poly(Ethylene-co-Butyl Acrylate) Composites for Electrical Applications
Source: Polymers (Basel). 2019 Apr 24;11(4):740. doi: 10.3390/polym11040740 (PMC6523082; doi:10.3390/polym11040740)
Supplement: Supplementary file 1 [file polymers-11-00740-s001.pdf]

Supporting information

Characterization of reduced and surface-modified graphene oxide in poly(ethylene-co-butyl acrylate) composites for electrical applications

Carmen Cobo Sánchez, Mattias Karlsson, Fritjof Nilsson, Martin Wåhlander, Henrik Hillborg, Eva Malmström*

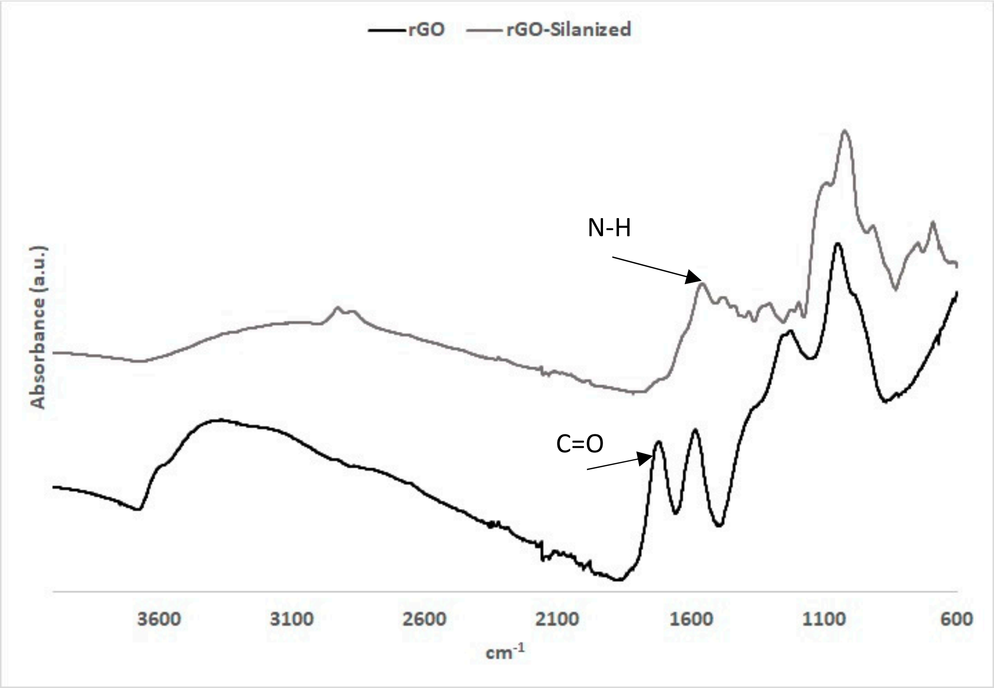

Figure S1. FT-IR spectra from the rGO and rGO-silanized samples. The peak for carbonyl bonds disappears for the rGO-silanized, appearing one more peak for the N-H bond from the APTES

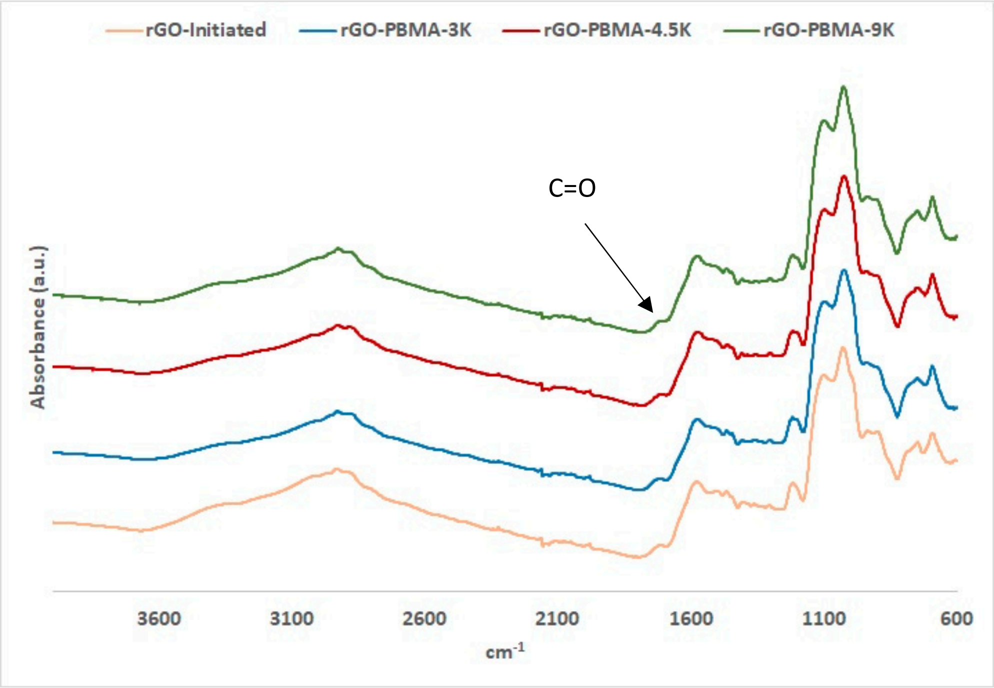

Figure S2. FT-IR spectra from the rGO-Initiated and the rGO-PBMAs. Due to the low quantity of polymer in the samples, no change in the carbonyl peak could be observed

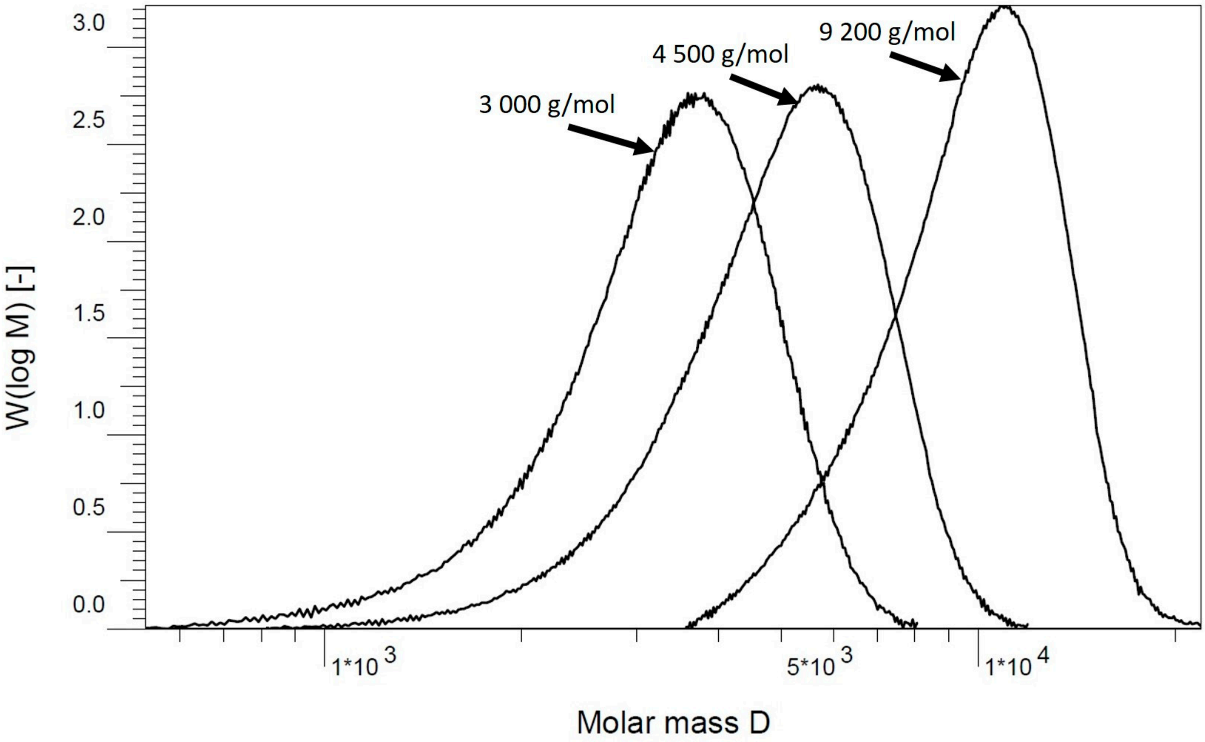

Figure S3. Overlay from the DMF-SEC samples measured from the polymer formed from the sacrificial initiator, corresponding from left to right to 2, 4 and 6 hours.

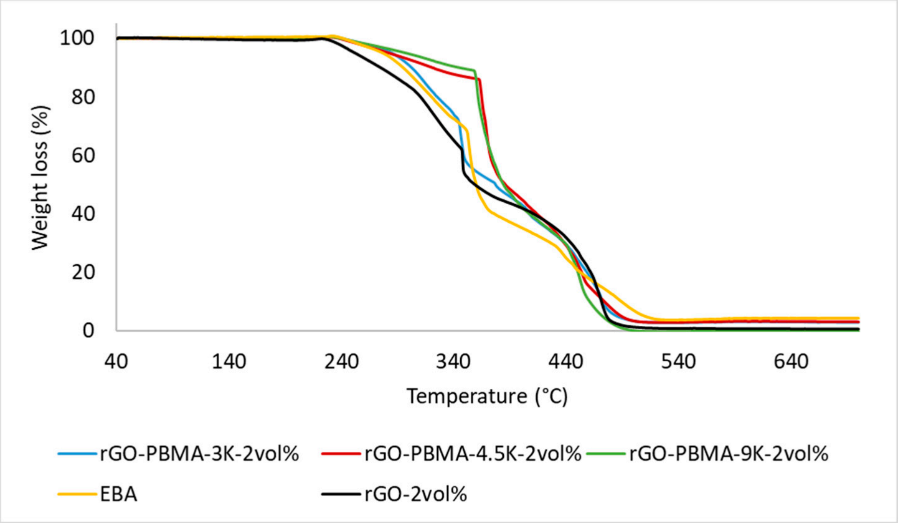

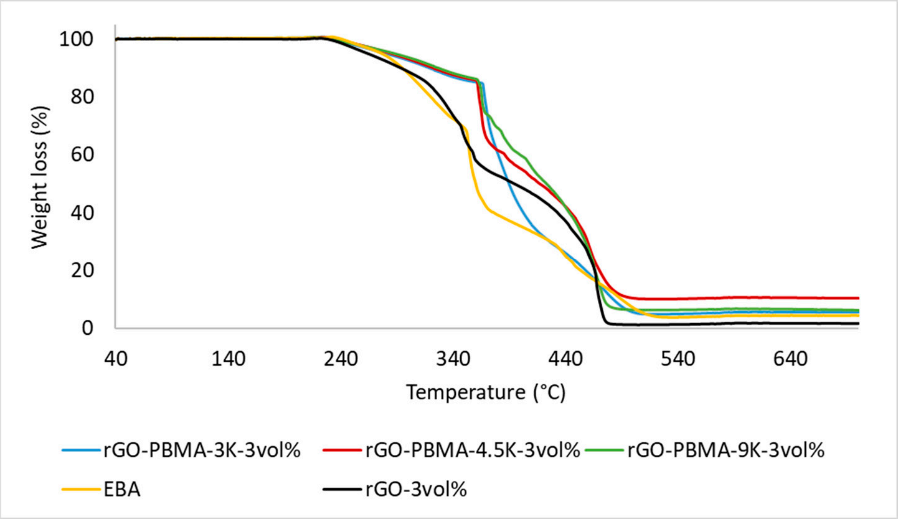

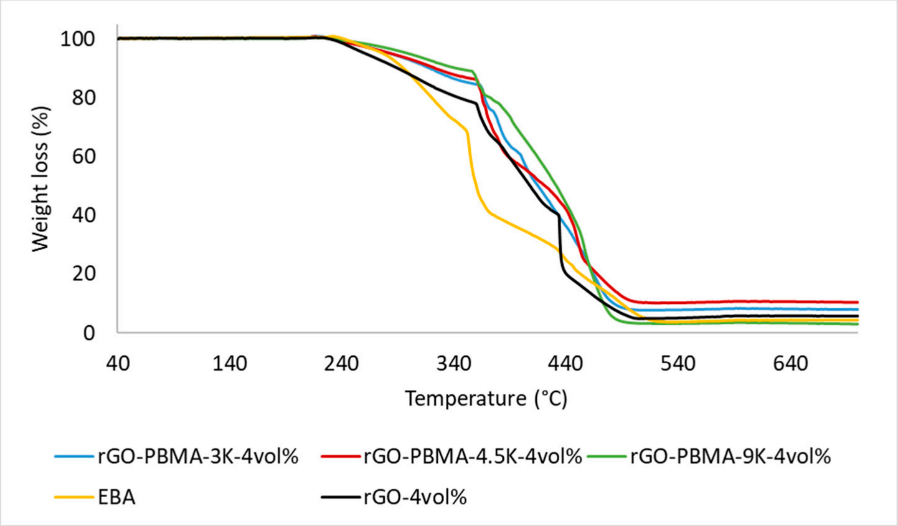

Figure S4. TGA thermograms for all the nanocomposites, up to down

	1st Heating			2nd Heating		Cooling	
	1st Peak	2nd Peak	Integral	Peak	Integral	Peak	Integral
EBA	42,5	91,6	10,13	91,6	9,92	76,4	10,29
rGO-2vol%	43,9	91,2	9,64	91,1	8,49	76,1	9,55
rGO-PBMA-3K-2vol%	44,5	92,1	10,37	91,4	9,01	76,8	9,54
rGO-PBMA-4.5K-2vol%	43,9	90,7	11,91	91,6	10,15	76,8	10,24
rGO-PBMA-9K-2vol%	44,4	91,1	10,43	91,7	8,55	76,6	9,39
rGO-3vol%	44,8	90,8	13,83	90,9	10,89	76,4	11,52
rGO-PBMA-3K-3vol%	43,9	90,6	10,16	91,3	9,83	76,1	9,74
rGO-PBMA-4.5K-3vol%	43,5	91,3	9,78	91,1	8,86	76,4	8,76
rGO-PBMA-9K-3vol%	43,6	91,6	10,62	91,1	9,04	76,4	9,11
rGO-4vol%	43,8	90,9	10,8	90,4	9,27	76,4	9,39
rGO-PBMA-3K-4vol%	41,6	91,3	11,26	91,3	9,03	76,1	10,24
rGO-PBMA-4.5K-4vol%	42	91,4	9,35	91,6	8,34	76,6	8,5
rGO-PBMA-9K-4vol%	42,3	91,6	11,01	91,3	9,91	76,6	9,96

Table S1. DSC data for all the nanocomposites

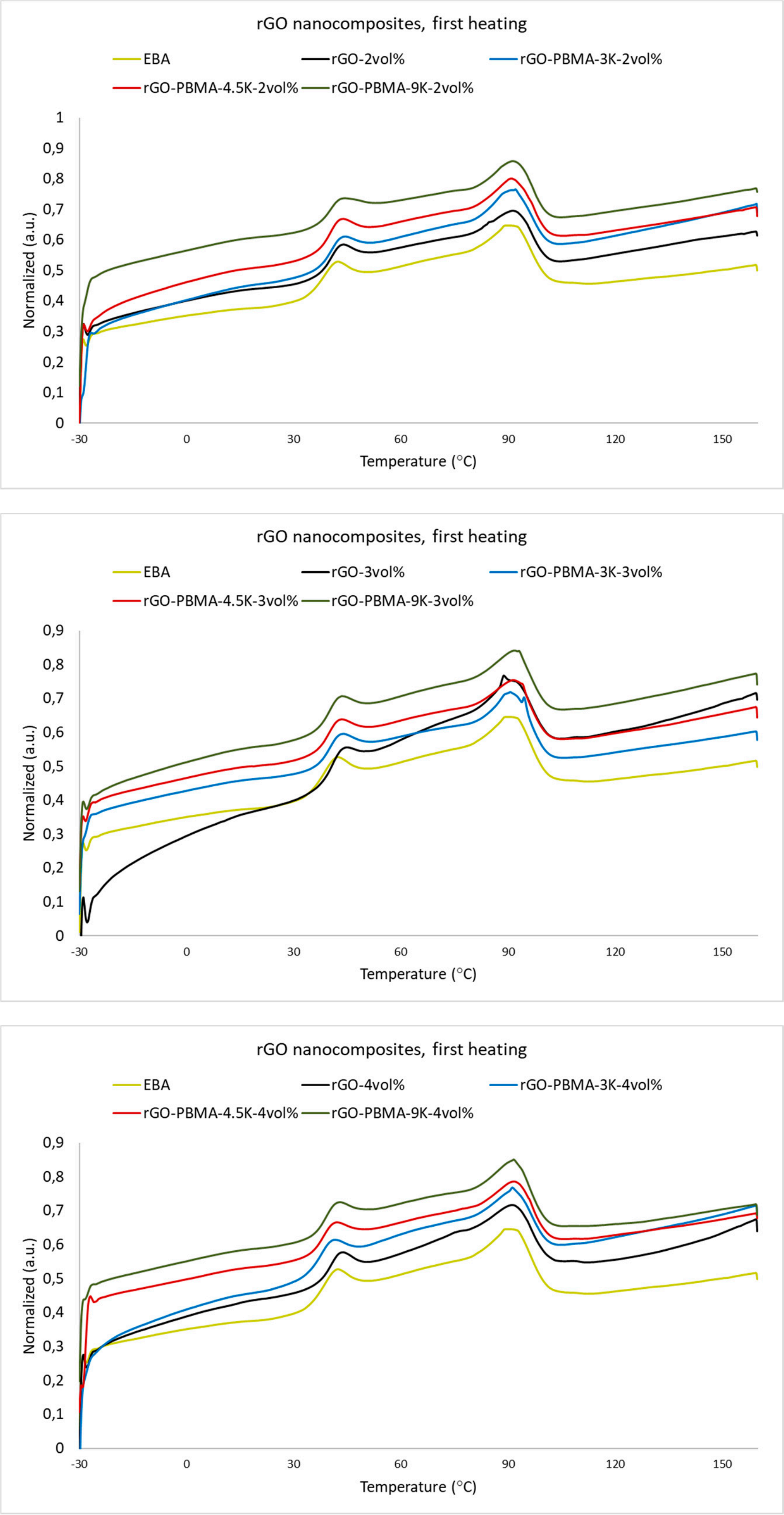

Figure S5. First heating thermograms for all the nanocomposites, up to down
